# Differences in the Phenolic Profile by UPLC Coupled to High Resolution Mass Spectrometry and Antioxidant Capacity of Two *Diospyros kaki* Varieties

**DOI:** 10.3390/antiox10010031

**Published:** 2020-12-30

**Authors:** Adelaida Esteban-Muñoz, Silvia Sánchez-Hernández, Cristina Samaniego-Sánchez, Rafael Giménez-Martínez, Manuel Olalla-Herrera

**Affiliations:** 1 Departamento de Nutrición y Bromatología, Universidad de Granada, 18071 Granada, Spain; silsanchez@ugr.es (S.S.-H.); csama@ugr.es (C.S.-S.); rafaelg@ugr.es (R.G.-M.); olalla@ugr.es (M.O.-H.); 2 Programme in Nutrition and Food Science, University of Granada, 18071 Granada, Spain; 3 Biosanitary Research Institute, IBS, 18071 Granada, Spain

**Keywords:** phenolic compounds, antioxidant capacity, UPLC-QTOF-MS, *Diospyros kaki*, persimmon, *Rojo Brillante*, *Triumph*

## Abstract

Background: phenolic compounds are bioactive chemical species derived from fruits and vegetables, with a plethora of healthy properties. In recent years, there has been a growing interest in persimmon (*Diospyros kaki* L.f.) due to the presence of many different classes of phenolic compounds. However, the analysis of individual phenolic compounds is difficult due to matrix interferences. Methods: the aim of this research was the evaluation of individual phenolic compounds and antioxidant capacity of the pulp of two varieties of persimmon (*Rojo Brillante* and *Triumph*) by an improved extraction procedure together with a UPLC-Q-TOF-MS platform. Results: the phenolic compounds composition of persimmon was characterized by the presence of hydroxybenzoic and hydroxycinnamic acids, hydroxybenzaldehydes, dihydrochalcones, tyrosols, flavanols, flavanones, and flavonols. A total of 31 compounds were identified and 17 compounds were quantified. Gallic acid was the predominant phenolic compounds found in the *Rojo Brillante* variety (0.953 mg/100 g) whereas the concentration of *p*-hydroxybenzoic acid was higher in the *Triumph* option (0.119 mg/100 g). Conclusions: the results showed that the *Rojo Brillante* variety had higher quantities of phenolic compounds than the *Triumph* example. These data could be used as reference in future phenolic compound databases when individual health effects of phenolic compounds become available.

## 1. Introduction

Persimmons (*Diospyros kaki* L.f.) belong to the *Ebenaceae* family and are a plant native to Asia, where 90% of the world’s production is located [1]. Persimmons have traditionally been used for juice or vinegar production [2,3,4], but also for medicinal purposes, to help the likes of cough relief, hypertension, dyspnea, paralysis, burns, and bleeding [5,6]. There are more than 1000 persimmon varieties differenced by astringency. Astringent persimmons are the most common class and are richer in tannins, while non-astringent varieties have less tannins, and are, therefore, the variety preferred by consumers [7,8]. The *Rojo Brillante* variety (astringent) is sweet at maturity but needs to be CO_2_ treated in order to reduce astringency [7]. On the contrary, the *Triumph* variety (non-astringent), mainly found in Andalusia (Spain), can be consumed as it is because the fruit matures on the tree [8,9]. However, the type of post-harvest treatment affects the final primary and secondary metabolite content of persimmon fruit with more bioactive compounds being found in astringent varieties than the non-astringent options [10].

Spain is the world’s second largest persimmon producer [1] and it is one of the crops with the highest growth rate within the country. In 2019, the harvest exceeded 400,000 tons, most of which was exported (70%), mainly thanks to the Kaki Ribera del Xúquer PDO (Protected Designation of Origin) variety (MAP 2019). On the contrary, the average consumption of this fruit in Europe is only 0.35 Kg/inhabitant compared to 24 Kg/inhabitant consumption of apples or pears.

Persimmons are rich in sugars and low in fats, while the composition of micronutrients, such as minerals, is interesting due to its high content in P and K [11]. Persimmons are also rich in many different types of phenolic compounds, which play a role in reducing different pathologies, such as diabetes, cardiovascular diseases, cancer, and Alzheimer’s disease [12,13,14,15,16]. Phenolic compounds are secondary metabolites of plant origin and considered the main antioxidant components in food [9,17], which could explain their healthy properties [18].

As far as we know, only a few studies have evaluated the profile of phenolic compounds in persimmon fruit [19,20,21,22,23]. The main analytical technique for the detection of these compounds is liquid chromatography (HPLC), coupled with a diode array detector [19], ultraviolet [24], fluorescence [6], and mass spectrometry [25]. For the purpose of this study, high-resolution mass spectrometry has been used to analyze of phenolic compounds in different fruits [26,27,28] due to its sensitivity-selectivity of detection and powerful structural elucidation of unknown phenolic compounds in complex samples. In addition, coupling of high-resolution platforms with chromatographic separation by ultra-performance liquid chromatography allows fast and reliable determination of more compounds in less time, minimizing the effect of coeluting interferences and increasing sensitivity [25]. However, such analytical platforms have not yet been used to analyze phenolic compounds in persimmon fruit. In this study a extraction technique coupled to an UPLC-QTOF-MS high-resolution mass spectrometer was used, having previously been tested by Esteban-Muñoz et al. [29] on other fruits.

Therefore, the aim of this study was to evaluate the antioxidant capacity and the individual phenolic compounds in two Spanish grown persimmon varieties (*Rojo Brillante* and *Triumph*), in order to evaluate and promote persimmon consumption as the most convenient functional food in the European diet compared to other more commonly consumed fruit. Moreover, the study aimed to update and complete the European food composition databases with indigenous products that more correctly reflect actual consumption.

## 2. Materials and Methods

### 2.1. Samples

Two varieties of persimmon were analyzed: *Rojo Brillante* and *Triumph*. The samples were obtained from various supermarkets in the cities of Granada and Malaga, from two different harvests. Each sample consisted of three different, randomly chosen pieces of fruit from either a basket or mesh packaging found within the fruit display. They were then immediately taken to the laboratory to be processed. All non-edible parts—skin, seeds, and/or pits—were removed; the separated edible part was then ground in a blender and the juice extracted from each fruit. The processed samples were stored at 4 °C until needed for analysis, which was performed as soon as possible. All analyses were conducted in triplicate.

### 2.2. Reagents

The following reagents were obtained from Sigma-Aldrich SL (Madrid, Spain): gallic acid, *p*-hydroxybenzoic acid, vanillic acid, protocatechuic acid, syringic acid, ellagic acid, pyrogallol, 3,4-dimethoxybenzoic acid, caffeic acid, dimethyl caffeic acid, *p*-coumaric acid, ferulic acid, 4-methoxycinnamic, sinapic acid, chlorogenic acid, tyrosol, and quercetin. Diethyl ether, acetic acid, anhydrous sodium sulfate, and methanol were from Panreac Química SL (Barcelona, Spain). Water was obtained from a Milli-Q purification system (Millipore, Bedford, MA, USA). Hydrogen peroxide (H_2_O_2_), anhydrous sodium carbonate (Na_2_CO_3_), hexane, and sodium hydroxide (NaOH) were all purchased from Carlo-Erba (Rodano, Milan, Italy). Methanol, Folin Ciocalteu reagent, 6-hydroxy-2,5,7,8-tetramethyl-chroman-2-carboxylic acid (Trolox), 2,2-azinobis-(3-ethylbensothiazoline)-6-sulfonic acid (ABTS), and 2,2-diphenyl-1-picrylhydrazyl (DPPH) were provided by Sigma-Aldrich (Milan, Italy) and potassium peroxodisulfate (K_2_S_2_O_8_) by Panreac (Barcelona, Spain). Gallic acid was obtained from Sigma (Milan, Italy). All reagents were of analytical grade.

### 2.3. Sample Treatment

**Figure 1:**
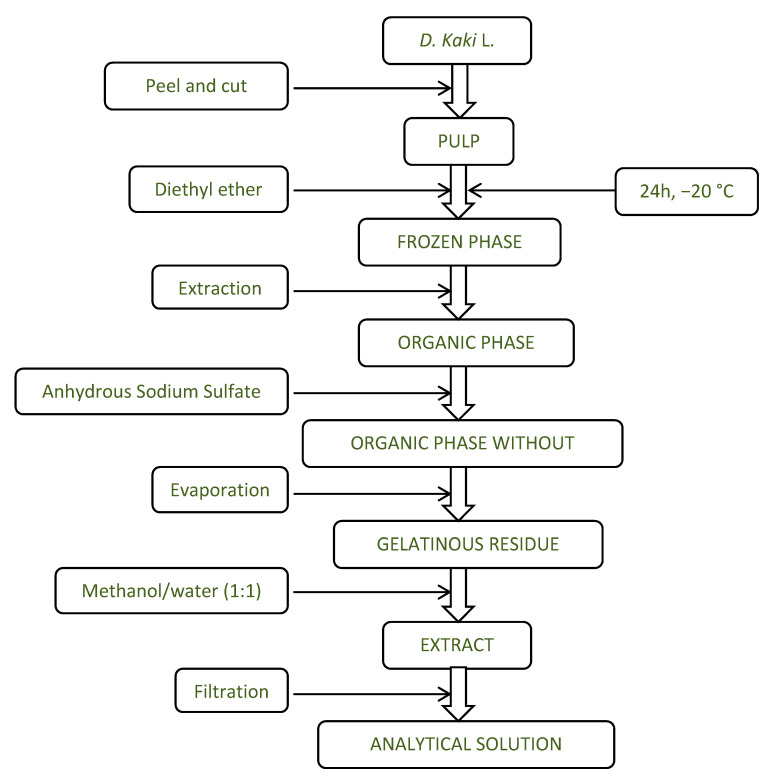
Schematic process of the extraction of phenolic compounds.

The method used for sample treatment was described by Moreno-Montoro et al. [30] with some changes described by Esteban-Muñoz et al. [29], which used diethyl ether instead of acidic methanol to extract phenolic compounds from different fruit matrixes. An amount of 20 mL of diethyl ether was briefly added to persimmon (100 g of homogenized pulp) and the resulting solution was frozen at −20 °C for 24 h. Then, the mixture was centrifuged for 10 min at 9000 rpm. The supernatant was transferred to a separatory funnel and three extractions were made with 20 mL diethyl ether. A spatula tip of anhydrous sodium sulfate was added to the organic extract, filtered, and evaporated under rotary evaporation at 30 °C to the smallest possible volume. The extracts were filled to 1 mL with a methanol/water mixture (1:1), filtered through a 0.20 μm membrane filter, and passed to the chromatography vial for analysis. The procedure is shown by Figure 1.

### 2.4. Individual Phenolic Compounds

An UPLC-QTOF-MS method [31,32] was used to identify the different polyphenols with some modifications described by Esteban-Muñoz et al. [29].

#### 2.4.1. Chromatographic and Mass Spectrometer Operating Conditions

The electrospray ionization (ESI)-MS experiments were performed on a liquid chromatography system with a hybrid mass spectrometer SYNAPT G2 HDMS Q-TOF model (Waters, Milford, CT, USA). The UPLC separation was performed using an ACQUITY UPLC™ HSS T3 2.1 × 100 mm, 1.8 µm C18 column (Waters, Milford, CT, USA). The program for chromatography was set with a binary gradient consisting of (A) water with 0.5% acetic acid and (B) acetonitrile, as follows: 0.0–15.0 min, 5% (B); 15.0–15.1 min, from 5 to 95% (B); and 15.1–18.0 min, from 95% to 5% (B), in this last part the column was reconditioned. Ten microliters of sample were injected and the flow rate was 0.4 mL/min. TOF conditions (measurement ranged from 50 to 1200 Da with the following mass spectrometer settings: nebulizer gas: 2 bar; drying temperature: 180 °C; capillary: 3100 V; drying gas: 6 L/min) consisted of a full MS and data-dependent scanning was performed in negative mode with electrospray ionization (ESI). The acquisition of fragment spectra for each mass was carried out using collision energy set to 40 eV with MS/MS mode.

#### 2.4.2. Identification and Quantification

Phenolic compounds were identified using the exact mass, isotopes, by comparing negative masses from previously recorded research and comparison of detected fragments ions with databases (ChemSpider, FooDB, and PubChem) using MassLynx V4 software (Waters, Milford, CT, USA) for instrument control, data acquisition, and data analysis. Individual phenolic compounds were quantified by obtaining a series of solutions, with a concentration of 0.1–40 mg/L of standards with different retention times. For each phenolic compound selected, we carried out a five-point calibration curve with R2 ≥ 0.93 in order to ensure the linearity of the method. The standards were analyzed under the same working conditions as the samples. An example of the identification of vanillic acid in a sample of *Rojo Brillante* is shown in Figure S1.

#### 2.4.3. Analytical Validation

Validation was made by studying the analytical parameters of selectivity, linearity, limit of detection (LOD), limit of quantification (LOQ), and precision according to the Association of Official Analytical Chemists´ guidelines for the validation of analytical methods [33]. The LOD and LOQ of the method were determined from the background response of three blanks using 1.0 mL of water. They were calculated as three (LOD) and 10 times (LOQ) the standard deviation (SD) of the response against the slope of the calibration curves for each phenolic compound. The intra-assay precision was evaluated by calculating the relative coefficient of variation (CV) of the analysis of 10 aliquots of each sample on the same day.

Figure S2a,b show an example of a chromatogram of *Rojo Brillante* and *Triumph* persimmon respectively. A total of 31 phenolic compounds were identified by UPLC-QTOF-MS in the crushed pulp of both persimmon varieties.

Table S1 reports the tested amount ranges, retention times, the adjusted linear equations, correlation coefficients, LOD and LOQ. All correlation coefficients were ≥0.99, except for 3,5-dimethoxybenzoic acid (*r* = 0.98), tyrosol (*r* = 0.96) and vanillic acid (*r* = 0.96). The LOD and LOQ (as mg/L) obtained are shown in Table S1. This method allowed the detection of 31 phenolic compounds and the quantification of 17 phenolic compounds at concentrations <6.4 and <21.3 mg/L for LOD and LOQ, respectively. The highest LOD and LOQ corresponded to 3,5-dimethoxybenzoic acid (6.377 and 21.258) and the lowest to sinapic acid (0.082 and 0.273). Intra-assay precision (% CV) is reported in Table S2. Precision was lower than 1.38 for all the phenolic compounds analyzed and ranged from 0.26% (*p*-coumaric acid) to 1.38% (*p*-hydroxybenzoic acid). The intra-assay precision values comply with those indicated by the AOAC (Association of Official Agricultural Chemists) [33] for a single laboratory (<4%).

### 2.5. Determination of Antioxidant Capacity

#### 2.5.1. Antioxidant Capacity (AC)

The AC of the samples was measured via three different methods, in a BMG Labtech FLUOstar Omega plate reader (Offenburg, Germany) using the Omega Control program and MARS Data Analysis Software (BMG LABTECH, Ortenberg, Germany). The DPPH scavenging assay was used as proposed by Cuvellier et al. [34]. The method derived the ferric reducing ability of FRAP according to the procedure described by Benzie and Strain [35] and the antioxidant equivalent capacity as radical scavenging activity, based on reduction of the radical cation ABTS assay following the procedure of Re et al. [36]. These assays were modified in our laboratory and these methods have been described in depth by our research group [37,38,39,40,41]. In all of the methods applied, we determined the dilution of persimmon methanolic extracts that gave a linear response. The absorbance signal was translated into antioxidant activity using Trolox as the external standard. Different calibration curve ranges were used depending on the method employed (Table S3). Results of the assays were expressed as micromoles of Trolox equivalents per gram (µmol TE/G). All experiments were conducted in triplicate and values expressed as averages ± standard deviation.

#### 2.5.2. Total Phenolics (TP)

The concentration of total phenolics was determined by the Folin–Ciocalteu colorimetric method described by Singleton and Rossi et al. [30] and modified in our laboratory [31]. We added 2.5 mL of deionized water and 500 µL of Folin–Ciocalteu reagent to 500 µL of methanolic pulp extract. The mixture was allowed to stand for 5 min, and then 2 mL of a 10% aqueous Na_2_CO_3_ solution was added. The final volume was adjusted to 10 mL. Samples and the blank were allowed to stand at room temperature for 90 min before being measured at 700 nm using a BOECO S-22 UV–VIS spectrophotometer (Hamburg, Germany). The amount of total phenolics is expressed as gallic acid equivalents per gram of fresh weight (mg GAE/g FW) through the calibration curve of gallic acid. The calibration curve range was 0.5–7.5 µg/mL (*r* = 0.991) (Table S3).

### 2.6. Statistical Analyses

Results are expressed as means ± SD. The SPSS 20.0 statistical package (IBM Inc., Armonk, New York, NY, USA) was used to analyze the data. Normality of variables was assessed by the Kolmogorov–Smirnov test (*p* < 0.05) to establish *p* the use of a parametric or non-parametric test for variance analysis. In addition, the Bartlett test (*p* < 0.05) was used to check the homogeneity of variances. As a result, the parametric Student’s *t*-test (95% significance level, established at *p* < 0.05) and the non-parametric Kruskal–Wallis test (95% significance level, established at *p* < 0.05) were carried out. Statistical analyses were conducted using GraphPad Prism 8.0.1 software (San Diego, CA, USA). The mean and standard deviation of values were determined, as were the differences between groups by means of a t-Student analysis and an analysis of variance (ANOVA). Differences with a *p* value < 0.05 were considered significant.

## 3. Results and Discussion

### 3.1. Individual Phenolic Compounds

#### 3.1.1. Qualitative Determination of Phenolic Compounds in Persimmon

Table S1 shows the 31 phenolic compounds identified by UPLC-QTOF-MS, including their molecular formula, mass retention times, the persimmon variety in which they were detected, theoretical, and experimental molecular mass and both errors in ppm, and the fragments. Compounds 1, 2, 3, 4, 5, 6, 7, 8, 9, 10, 11, 13, and 26 of Table 1 were identified by comparing with authentic standards that were later used in quantification; those that were not quantified with authentic standards were identified by comparing with fragmentation standards and previous publications. The phenolic compounds compositions of the *Rojo Brillante* and *Triumph* varieties were characterized mainly by the presence of hydroxybenzoic acids, hydroxycinnamic acids, hydroxybenzaldehydes, tyrosols, dihydrochalcones, flavanols, flavanones, and flavonols.

Other literature mentions the presence of simple phenolic compounds (hydroxybenzoic acids and hydroxycinnamic acids), tannins, procyanidins, flavonoids, and tyrosols [20,25,42] in persimmon fruit. Among hydroxybenzoic acids compounds, Table 1 shows that gallic acid was detected in both varieties, with a characteristic (M-H)]—experimental at *m*/*z* of 169.0139 and 169.0135 in *Triumph* and *Rojo Brillante* samples, respectively. The peak at 2.55 min in both extracts showed an (M-H)—experimental at *m*/*z* of 137.0239 and a molecular formula corresponding to *p*-hydroxybenzoic acid. Regarding hydroxycinnamic acids, major compounds *p*-coumaric, and caffeic acids were identified in both *Rojo Brillante* and *Triumph* samples, by means of distinctive (M-H)—experimental at *m*/*z* 163.0401 and *m*/*z* 179.0336, respectively. Figure S1 shows an example of a chromatogram of a *Rojo Brillante* sample, chromatogram extracted from vanillic acid, and a spectrum used for identification.

#### 3.1.2. Quantitative Determination of Phenolic Compounds in Persimmon

In this study, the phenolic compounds species quantified by UPLC-QTOF-MS were seven hydroxybenzoic acids (gallic, vanillic, *p*-hydroxybenzoic, protocatechuic, syringic, ellagic and 3,5-dimethoxybenzoic acids) and seven hydroxycinnamic acids (caffeic, dimethyl caffeic, ferulic, *p*-coumaric, 4-methoxycinnamic, sinapic, and chlorogenic acid). All of these phenolic compounds were non-flavonoid phenols and other phenolic compounds such as pyrogallol. We also detected and quantified tyrosol and a flavonoid species (the flavonol quercetin). Table 2 shows the concentrations (mg/100 g fruit pulp) of the 17 phenolic compounds quantified in samples of *Triumph* (*n* = 10) and *Rojo Brillante* (*n* = 10) persimmon varieties.

##### Hydroxybenzoic Acids

There were seven hydroxybenzoic acids among the non-flavonoid phenolic compounds (Table 2). In this study, gallic acid was the most predominant phenolic compound found in extracts of the *Rojo Brillante* variety (0.953 ± 0.344 mg/100 g fruit pulp), whereas in *Triumph* extracts, the concentration was 30 times lower (*p* < 0.05). These values are in line with those reported by other authors [21,43], but below the concentrations stated by Gao et al. and Fu et al. [22,23]. On the contrary, some authors did not detect gallic acid in persimmon pulp, in varieties such as *D. kaki Jiro* and *D. kaki Zenjimaru* [21,44]. According to Macheix et al. [45], gallic acid is found in many fruits and vegetables in the range of 0.5–15 mg/100 g. Gallic acid can exert beneficial health effects, especially important for its anticarcinogenic and cardioprotective activities [46,47]. In this sense, in-vitro studies have shown gallic acid to have great effectiveness against human prostate cancer [48] and digestive cancers [49].

The levels of *p*-hydroxybenzoic acid were similar for the *Triumph* and *Rojo Brillante* varieties (Table 2). These results are in line with the observations of other authors regarding the presence of *p*-hydroxybenzoic acid in persimmon leaves [25] or different varieties like *D. kaki Zenjimaru* and *D. kaki Zhouqumomoshi* [21], but 2–7 lower than other varieties [21,43]. Higher concentrations of this phenolic compound have been reported in other fruit pulps, such as white grapes [30]. Moreover, *p*-hydroxybenzoic acid is of interest to agriculture as it regulates antioxidant enzyme activity and mitigates heat stress in cucumber leaves [50].

Vanillic acid is a very common phenolic compound that can be added to foods as flavoring and an olfactory agent giving a pleasant and creamy aroma [51]. It is an intermediate product in the two-stage bioconversion of ferulic acid to vanillin [52], and it selectively inhibits 5‘nucleotidase activity [53]. It was identified in the two varieties of persimmon, with a statistically higher concentration (*p* < 0.05) in *Rojo Brillante* (0.056 ± 0.013) than in *Triumph* (0.021 ± 0.016). The same tendency was observed for protocatechuic acid (Table 2) as the concentrations of the *Rojo Brillante* variety (0.013 mg/100 g) were higher (*p* < 0.05) than those obtained for the *Triumph* sample (0.004 mg/100 g). Higher concentrations were found in different persimmon extract obtained with different solvents [22], but the use of an HPLC equipped with ECD (electron capture detector) detection could overestimate the concentration. Protocatechuic acid has been demonstrated as an effective antiproliferative compound on HL-60 cells due to apoptotic effects [54].

Syringic and ellagic acids were detected and quantified only in the *Rojo Brillante* variety (0.037 and 0.327 mg/100 g fruit pulp, respectively). In the case of syringic acid, the results are in line with those reported for other varieties (Table 2), ranging from 0.07 to 0.32 mg/100 g [21]. It has been reported as a product of the microbial metabolism of anthocyanins and other phenolic compounds [55] related to the positive role of wine phenolic compounds against oxidation of low-density lipoprotein [56]. On the other hand, to the best of our knowledge, this is the first time ellagic acid has been identified in persimmon. It is a phenolic compounds with interesting antioxidant, estrogenic and/or antiestrogenic, anti-inflammatory, antimicrobial, and prebiotic effects [57].

##### Hydroxycinnamic Acids

Among the non-flavonoid phenolic compounds, seven hydroxycinnamic acids were identified and quantified. The second most abundant hydroxycinnamic phenolic compound in both varieties was caffeic acid, with 0.078 and 0.046 mg/100 g for the *Triumph* and *Rojo Brillante* varieties, respectively (Table 2). These levels are in line with those reported for other persimmon varieties [21,22]. On the contrary, there are no data available about the levels of dimethylcaffeic acid concentrations in persimmon, which was quantified only in the *Rojo Brillante* variety (0.022 mg/100 g). Caffeic acid has been described as a potent antioxidant and reducing species compared to other antioxidants like coumaric or ferulic acids, BHA (butylhydroxyanisole), BHT (butylhydroxytoluene) or α-tocopherol (Gulcin, n.d.), and has also shown to have a protective effect against ultraviolet radiation [58].

*p*-coumaric acid was the hydroxycinnamic acid with highest concentrations in both varieties of persimmon (not statistically different), ranging from 0.088 to 0.113 mg/100 g fruit pulp. These values are slightly higher (Table 2) than those reported by other authors [21,22].

Ferulic acid is a natural antioxidant abundant in the cell walls of plants and is commonly found in fruit and vegetables [59]. It has been described as having many physiological functions, including antimicrobial, anti-inflammatory, anti-thrombosis, and anticarcinogenic effects [60]. As stated in Table 2, *Rojo Brillante* persimmon had a statistically higher (*p* < 0.05) concentration of ferulic acid than the *Triumph* variety (0.011 vs. 0.008 mg/100 g). These levels are in line with (or slightly lower than) those reported for different persimmon varieties by other authors [21,43] (Table 2).

In the case of 4-methoxycinnamic and sinapic acids, their levels (Table 2) were quite low (0.001–0.002 mg/100 g fruit pulp) but this is the first time that these phenolic compounds have been found in persimmon (Table 2). In a recent study by Zare et al. [61], it was shown that sinapic acid has neuroprotective potential in Parkinson’s disease in rats.

Finally, chlorogenic acid was only quantified in the *Triumph* variety (0.007 mg/100 g). Some authors have detected chlorogenic acids at higher concentrations in other varieties of persimmon [21], whereas other authors have not detected it at all in different types of persimmon pulp [43]. Chlorogenic acid anticarcinogenic activity in rats is due to its high antioxidant activity [62].

##### Tyrosols

Tyrosol is a natural antioxidant with the effect of preventing protein damage caused by hydrogen peroxide but not the superoxide anion released during the respiratory burst [63]. Tyrosol was detected at similar levels in both persimmon varieties (Table 2), but no other studies discussing tyrosol have been found.

##### Flavonols

According to Macheix et al. [45], the usual range of flavonols in fruits is 0.5–25 mg/100 g. As a result, it is expected that persimmon would have flavonols in its chemical composition. In general, the extracts of other varieties of persimmon described gallic acid and quercetin as the most predominant phenolic compounds [21]. Quercetin was quantified in similar proportions in the *Triumph* and *Rojo Brillante* varieties (Table 2) in the range of 0.005–0.007 mg/100 g fruit pulp. Although these values are lower than those reported for other persimmon varieties [21], some other authors did not detect any quercetin in persimmon [43]. Quercetin has been reported to effectively recycle vitamin E and is also known to reduce inflammation, tumorigenesis, and cell damage caused by oxidation [64].

##### Other

Pyrogallol was quantified in both persimmon varieties, being higher in the *Rojo Brillante* variety (*p* < 0.05). The presence of pyrogallol in persimmon has not been previously reported in other papers, but it was found in other persimmon species, such as Diospyros Castanea. Its crude extract exerted high toxicity and selectivity against HepG2 and HeLa cells due to the production of intracellular superoxide anion cells [4,48]. The levels of 3,5-dimethoxybenzoic acid were similar in both the *Triumph* and *Rojo Brillante* varieties.

Comparing these values (using a simulate analytical method with other studies of our group) [29] mainly with tropical fruits, much higher levels of hydroxybenzoic acids and especially gallic acid (0.953 mg/100 g) are clearly observed in the *Rojo Brillante* variety. Three types of fresh fruit were compared with this variety of persimmon and were found to have compatible high tannin content (epicatechin gallate and epigallocatechin gallate): cherimoya 0.0735 mg/100 g, papaya 0.0341 mg/100 g, and pitaya 0.0546 mg/100 g. These compounds are commonly called catechins and form a group of bioactive compounds with strong antioxidant capacity involved in many oxidative stress-based chronic diseases.

The effects of persimmon on human lymphoid leukemia Molt 4B cells have also been studied. In this sense, a persimmon extract and some of its individual polyphenols (catechin, epicatechin, epigallocatechin and epicatechin gallate) were investigated [65]. These authors found that persimmon extract, as well as epigallocatechin and epicatechin gallate inhibited the growth of these cells in a dose dependent manner. After three days of treatment, they observed severe cell damage, such as DNA fragmentation. These findings could indicate a possible use for therapeutic purposes. Additionally, as part of a case-controlled study of thyroid cancer in Korean women, an inverse correlation was found between persimmon consumption and malignant, as well as benign, thyroid cancer risk [66].

### 3.2. Determination of Antioxidant Activity

#### 3.2.1. Antioxidant Capacity (AC)

**Figure 2:**
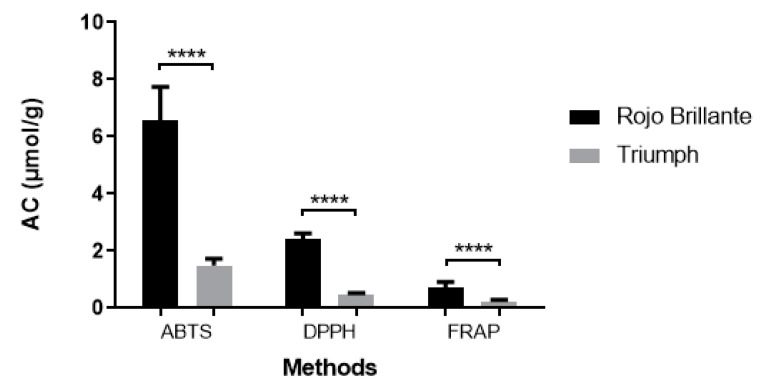
Evaluation of the antioxidant activity (AC) of the *Rojo Brillante* and *Triumph* varieties measured by ABTS, DPPH, and FRAP assay. **** Indicates *p* < 0.0001.

The ABTS and DPPH radical scavenging activities and FRAP ferric reducing ability of the persimmon varieties were determined, and the results are shown in Table 3. Figure 2 summarizes the antioxidant capacities of persimmon measured by the different methods. The antioxidant activity values expressed in micromoles Trolox per gram ranged from 1.280 ± 0.069 to 8.865 ± 0.056 μmol TE/g when measured by ABTS, 0.458 ± 0.05 to 2.633 ± 0.03 μmol TE/g when measured by DPPH, and 0.206 ± 0.01 to 0.965 ± 0.005 μmol TE/g when the FRAP method was used.

The greatest ABTS·+ scavenging capacity was detected in *Rojo Brillante* (6.572 μmol TE/g), while the lowest was found in the non-astringent variety *Triumph* (41.484 μmol TE/g). The ABTS·+ scavenging capacity in *Rojo Brillante* was almost six fold higher than that of the *Triumph*.

The radical scavenging of varieties on DPPH (Table 3) showed similar diversity to the results of the ABTS·+ assay, with the astringent variety almost twofold higher than the non-astringent variety. The antioxidant activities of all extracts determined as DPPH radical scavenging ability ranged from 2.633 to 0.458 μmol TE/g.

The ferric reducing antioxidant power of *Rojo Brillante* and *Triumph* extracts was similar to the DPPH scavenging activity and ABTS·+ scavenging capacity, with higher values for the astringent variety (0.965 ± 0.005 μmol TE/g).

In general, the astringent variety (*Rojo Brillante*) showed much higher antioxidant activity than the non-astringent variety (*Triumph*), for both ABTS (6.572 µmol TE/g and 1.484 µmol TE/g, respectively); DPPH (2.417 µmol TE/g and 0.492 µmol TE/G, respectively) and FRAP assay (0.731 µmol TE/g and 0.242 µmol TE/g, respectively). The *Rojo Brillante* variety also has the highest values of total phenol content as measured by the Folin method (380.786 µg GAE/g and 81.568 786 µg GAE/g, respectively) (Table 3).

For persimmon samples, these results were similar to those of other authors [67]. Their results ranged from 1027.03 to 1667.65 µmol/kg), concentrations very close to those reported in our study, although very few papers discussed antioxidants for the fruits considered in this study. Our results are similar and consistent with data from research, which investigated diverse persimmon genotypes.

Li et al. reported much higher values for the ABTS and DPPH assays in the astringent variety than the non-astringent option, for both pulp and peel. However, the FRAP assay did not show any great difference as in our study. The pulp obtained higher values in the astringent variety rather than the non-astringent option while the opposite occurred in the peel [68]. Lucas-González et al. also reported values higher by ABTS, DPPH, and FRAP assays for *Rojo Brillante* than *Triumph* persimmon [10].

Two authors determined AC with ABTS, DPPH, and FRAP assays. Loizo et al. determined AC in a variety of astringent persimmon *Diospyros lotus* L., (72.6 ± 1.5 µg/mL for DPPH, 0.21 ± 0.07 µM/g for FRAP and 0.36 ± 0.01 TEAC for the ABTS method) [69]. Pu et al. obtained higher AC values in the astringent varieties for ABTS (648.79, 889.14, and 1110.13 µmol/100 g); DPPH (898.91, 1588.26, and 1209.13 µmol/100 g) and FRAP assay (354.61, 699.43, and 613.82 µmol/100 g) than for the non-astringent varieties by three methods (47.86 and 106.59 µmol/100 g; 190.83 and 560.77 µmol/100 g; 111.78 and 90.10 µmol/100 g, respectively) [21]; persimmon-based dairy products activity was measured by DPPH. Their results ranged from 1027.03 to 1667.65 µmol/kg), concentrations very close to those reported in our study

Chen et al. determined antioxidant activity by using the ABTS and DPPH methods. The *Mopan* persimmon extract exhibited the strongest inhibiting activities against both ABTS (23.512 μmol/g) and DPPH (22.485 μmol/g) radicals [70]. This study did not find great differences between the two methods like our study did; but it did report higher values than in ours. In another study, in which AC was measured by the three methods, results contrary to ours were determined, obtaining higher AC values in non-astringent varieties (*Triumph*) than in an astringent option (*Rojo Brillante*) [71].

Furthermore, tannin concentration is one of the major antioxidants in persimmon pulp and this is where astringent and non-astringent persimmons differ the most. The greater presence of tannins in the astringent varieties could explain why higher AC can be found in them rather than in non-astringent varieties [72].

The variations found between the different studies may be due to the fact that antioxidant properties of fruit are strongly affected by the species, variety, and cultivation conditions of the particular plant [73].

Results of this study showed that the astringent persimmon extracts had stronger ABTS, DPPH radical scavenging activities, and ferric reducing antioxidant power, than the non-astringent extract. The diversity of ABTS·+ and DPPH radical scavenging activities could be explained by the different stoichiometry reactions between fruits and the two radicals. Moreover, the antioxidant properties of fruit greatly depend on the type of fruit (species and variety within species), and cultivation conditions (environmental and cultivation techniques) [73].

Our results also show that the antioxidant activity of persimmon can be significantly correlated with total phenolic compounds. In this sense, the greater capacity of *Rojo Brillante* variety can be attributed to having more individual phenolic compounds than the *Triumph* variety, as it has, regardless of the method used, a lower total, and individual phenolic compounds content, and a lower antioxidant activity. Other authors, such as Chen et al., have also described this relationship, which showed a positive and highly significant correlation between total phenolics content and radical scavenging activity against ABTS and DPPH radicals [70].

According to the aforementioned study on tropical fruits [29] the antioxidant capacity for the *Rojo Brillante* variety, measured by the ABTS method, was superior to avocado (5455 µmol/g), mango (4889 µmol/g), papaya (4221 µmol/g) and carambola (2271 µmol/g) but lower than that of cherimoya (52,142 µmol/g), and kiwi (45,627 µmol/g). According to Park et al. (2015), the antioxidant capacity of persimmon is higher than that of banana, grape, grapefruit, and lemon, but lower than that of strawberry, apple pulp, kiwi, and mangosteen [44].

With reference to other widely consumed fruits, according to the data of Fu et al., using the ABTS method [74], where all values are expressed as µmol Trolox/g fruit, the *Rojo Brillante* variety of persimmon presents a higher antioxidant capacity than apple (36.0%), banana (176.0%), grape (168.2%), peach (176,%), and pear (178.4%). Likewise and according to Scalzo et al., again using the same method (ABTS) and units, the *Rojo Brillante* variety presents a higher antioxidant capacity (6.572 µmol/g) than apple (1.60 µmol/g), apricot (1.39 µmol/g) or peach (1.22 µmol/g), with only strawberry (113.17) presenting less [73].

To a certain extent, this high antioxidant capacity, together with the functional activities already mentioned, also highlights diabetes prevention, or reduces oxidative damage caused by diabetes when free radicals are released. Furthermore, due to its high antioxidant content, the persimmon could help prevent or reduce LDL oxidation, and, thus, the development of atherosclerosis [75]. Additionally, tannins have shown the ability to trap bile acids, which could lead to a reduction in plasma cholesterol levels, resulting in a reduction of cholesterol levels and risk of cardiovascular disease [76].

#### 3.2.2. Total Phenolics (TP)

The total phenolics in both persimmon varieties were evaluated with the Folin–Ciocalteu method. *Rojo Brillante* samples showed high total phenol contents ranging from 202.163 μg/g to 532.089 μg/g gallic acid (µg GAE/g). The lowest total phenolic contents (ranging from 41.070 to 116.073 μg GAE/g) were found in the *Triumph* sample, which also has the lowest values for antioxidant capacity as measured by the direct methods (Table 3).

**Figure 3:**
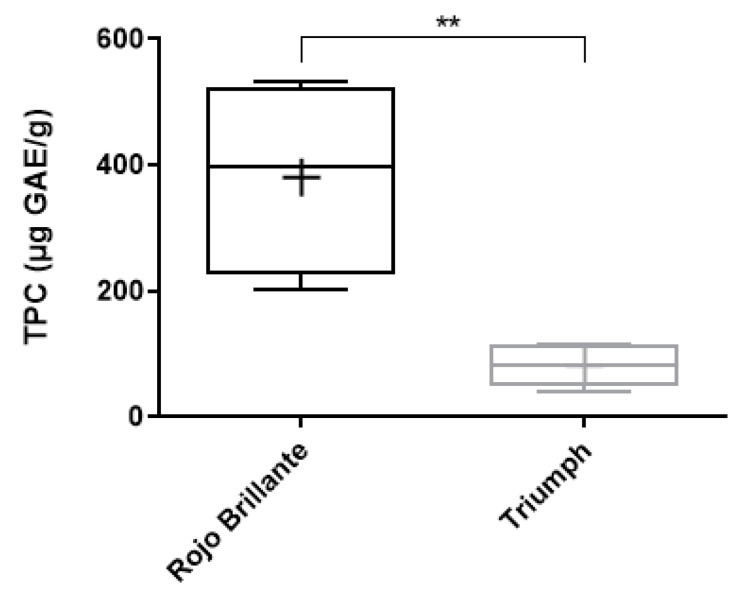
Quantification of the total phenolic compounds (TPC) in µg GAE/g of two persimmon fruit varieties (*Rojo Brillante* and *Triumph*). ** Indicates *p* < 0.01.

Thus, the *Rojo Brillante* variety (380.786 ± 158.539 µg GAE/g) showed a significantly (*p* < 0.01) higher TP than the *Triumph* variety (84.568 ± 34.989 µg GAE/g.), as shown in Figure 3. It has been reported that the TP may vary in different persimmon varieties and can vary significantly depending on the ripening stage or the astringency of the specific cultivar studied [77,78].

Similar or higher values have been reported in the other scientific literature; however, any information regarding TP is rather scarce. A recent study reported similar results, with a TP (mg GAE/100 g FW) of 59.2 ± 0.4 [79].

In the study of Suzuki et al., the TP of three astringent persimmons (HN; 84.6, TW; 68.3 and IW; 76.4 mg/100 g) were four to six times higher than those of two non-astringent persimmons (MJ; 18.4 and MF; 14.8 mg/100 g) [80], presenting very similar values to those of our study. According to the study by Lucas-González et al., 2018 [10], total phenolic compound (TPC) was also higher in the *Rojo Brillante* than the *Triumph* variety. Ercisli et al. (2008) reported a similar variation of TP from 157 to 423 mg GAE/g, and differences were observed among persimmon genotypes [81].

Li et al. reported higher TP values for the astringent variety of persimmon, both for flesh and peel samples, than the non-astringent variety [68]. Lucas-González et al. determined TP in both the *Rojo Brillante* and *Triumph* varieties (undigested, oral digested, gastric digested, and intestinal digested samples). Undigested samples presented the same values in both varieties (≈100 mg/g) [71].

Veberic et al. determined TP in 11 cultivars of persimmon fruit, three astringent varieties (*Fuji, ToneWase,* and *Triumph*) and eight non-astringent varieties (*Amankaki*, *Cal Fuyu*, *Hana Fuyu*, *Jiro*, *O’Gosho*, *Tenjin O’Gosho*, *Thiene*, and *Tipo*). The *Fuji* variety obtained the highest TP concentration (295 ± 31.3 mg GAE/kg). However, the other two astringent varieties, including the *Triumph* variety (127 ± 17.9 mg GAE/kg) did not present higher values with respect to the rest of the non-astringent varieties [20].

Pu et al. also reported higher concentrations of TP for the astringent varieties analyzed (538.30 mg GAE/100 g for *D. kaki. Cv. Xingyangshuishi*; 905.53 mg GAE/100 g for *D. kaki. Cv. Zhouqumomoshi* and 1055.02 mg GAE/100 g for *D. kaki. Cv. Xiuningbianshi*) than the non-astringent varieties (168.22 mg GAE/100 g for *D. kaki. Cv. Jiro* and 347.77 mg GAE/100 g for *D. kaki. Cv. Zenjimaru*) [21]. Nevertheless, these authors obtained higher TP content than our study.

Chen et al. determined total amounts of phenolic in the astringent persimmons (*Diospyros kaki L. Cv. Mopan*) and other fruits (grape, apple, and tomato). The *Mopan* persimmon showed the highest TP (168.15 ± 0.12 mg GAE/100 g) [19]. In another study that determined TP in 62 fruits, persimmon was one of the fruits with the highest content of these compounds [23] showing a value of 112.09 ± 4.60 mg/100 g. However, both these studies reported three times higher values than the varieties in our study.

In summary, this study showed that astringent persimmons (*Rojo Brillante*) has significantly higher concentrations of total phenolic and greater capacities than non-astringent persimmons (*Triumph*).

### 3.3. Nutritional Impact of Persimmon as an Antioxidant

In recent years, there has been increasing interest in dietary phytochemicals including isothiocyanates, phenolic compounds, flavonoids, isoflavones, lignans, saponins and coumestrol—due to their possible protective action against cardio A. vascular disease and certain types of cancer [82].

A persimmon´s functional properties are related to its bioactive compounds content, which exerts a protective role on hypercholesterolemia, diabetes, cancer, hypertension and some dermic disorders [44]. In a wider sense, the functional properties of persimmon have been related to its antioxidant capacity. For example, a randomized controlled trial studied the plasmatic antioxidant capacity and urinary excretion of 8-isoprostane in individuals with a high intake of persimmon vinegar [82]. A significant increase in plasma antioxidant activity and reduced excretion of 8-isoprostane (a urinary biomarker of oxidative stress) were found.

To try to establish the nutritional value of introducing persimmon consumption into the Spanish diet, some estimates were made regarding polyphenolic compounds intake according to the studies by Saura-Calixto et al. They estimated its intake at 1209 mg/person/day, these being the main source of exogenous antioxidants [83]. From the consumption data, Román Martínez et al. calculated the total antioxidant capacity (TAC) of the diet as 10,577.9 micromoles of Trolox equivalent (water-soluble vitamin E analog) per day. Based on the previous results, fruit (the group with the highest contribution) [84] contributes 37.20% of the total.

A daily intake of persimmon (200–400 g) over a period of 3 to 5 months of the year would suppose 300 g/day × 6.57 µg Trolox/g = 1971 µg Trolox/day = 18.60% of the total dietary antioxidant capacity intake, with approximately 50% of that contributed by the fruit.

According to the ORAC method (Román Martínez et al., 2012), which is based on real consumption (kg/person/day), this contribution is considerably higher than the values in μmol Trolox/day of CAT (total antioxidant capacity) of more commonly consumed fruits in the Spanish diet (MAP 2018): apple (690.54.70), pear (261.81), orange (846.86), banana (282.95), peach (156.6), grape (72.49), melon (65.13), and watermelon (30.42) [84,85].

A joint FAO/WHO expert consultation published a report recommending a minimum consumption of 400 g of fruit and vegetables per day (excluding starchy tubers, such as potatoes) to prevent chronic diseases, particularly heart disease, cancer, type 2 diabetes, and obesity.

Both organizations agree that consuming a wide variety of fruit and vegetables helps ensure sufficient intake of most micronutrients, dietary fiber, and other health beneficial non-nutrient substances. A higher consumption of fruit and vegetables can also help replace excessive consumption of less healthy foods (e.g., those rich in fat, sugar, or salt) [1].

## 4. Conclusions

The aforementioned UPLC-Q-TOF-MS adequately described the phenolic composition of two *Diospyros kaki* varieties (*Triumph* and *Rojo Brillante*). These fruits were characterized by the presence of hydroxybenzoic and hydroxycinnamic acids, flavanols, flavonols, flavones, flavanones, tyrosols, and other compounds. Using the UPLC-Q-TOF-MS platform, a total of 31 different phenolic compounds were identified, of which 17 were quantified. The results show the *Rojo Brillante* variety is richer in many different phenolic compounds with bioactive properties. Therefore, the potential beneficial effects of persimmon, especially *Rojo Brillante*, could be evaluated in the future.

Furthermore, this study showed that astringent persimmons (*Rojo Brillante*) had significantly higher concentrations of phenolic compounds and greater capacities than non-astringent persimmons (*Triumph*). Daily persimmon consumption would provide a greater amount of phenolic compounds (catechins) and a higher antioxidant capacity (taking into account the current consumption/person) than the majority of fruits consumed by most. This makes us propose it as a functional food to improve oxidative stress levels and the current high rates of cardiovascular disease, diabetes, or cancer in the general public.

## Figures and Tables

**Table 1:** Individual phenolic compounds identified in *Rojo Brillante* and *Triumph*.

No.	Proposed Compounds	Molecular Formula	R_t_ (min)	Extract	(M-H)^−^ calc.	(M-H)^−^ exp.	Error (ppm)	MS Fragments
	Phenolic acids/ Hydroxybenzoic Acids							
1	Gallic acid	C_7_H_6_O_5_	1.24	*Triumph*	169.0137	169.0139	1.0	125.0229, 123.0071
				*Rojo Brillante*	169.0137	169.0135	1.0	125.0236, 123.0062, 107.0111
2	Vanillic acid	C_8_H_8_O_4_	2.88	*Triumph*	167.0344	167.0345	0.1	153.0145, 109.0310
				*Rojo Brillante*	167.0344	167.0340	2.9	121.0285
3	*p*-Hydroxybenzoic acid	C_7_H_6_O_3_	2.53	*Triumph*	137.0239	137.0243	2.9	121.0268
				*Rojo Brillante*	137.0239	137.0240	0.1	121.0270
4	Protocatechuic acid	C_7_H_6_O_4_	1.89	*Triumph*	153.0179	153.0188	2.0	109.0343
				*Rojo Brillante*	153.0179	153.0199	2.6	109.0343, 107.0051
5	3,4-Dimethoxybenzoic acid	C_9_H_10_O_4_	5.50	*Triumph*	181.0507	181.0501	1.1	121.0241, 107.0143
				*Rojo Brillante*	181.0507	181.0496	3.9	137.0605
	Phenolic acids/Hydroxycinnamic Acids							
6	Caffeic acid	C_9_H_8_O_4_	2.85	*Triumph*	179.0344	179.0351	3.7	135.0462, 133.0267, 123.0416
				*Rojo Brillante*	179.0344	179.0342	1.2	135.0462, 121.0235, 107.0155
7	Dimethyl caffeic acid	C_11_H_12_O_4_	5.02	*Rojo Brillante*	207.0657	207.0659	1.0	191.0306, 163.0337, 133.0658
8	Ferulic acid	C_10_H_10_O_4_	3.90	*Triumph*	193.0501	193.0502	0.8	147.0396, 133.0295, 105.0355
				*Rojo Brillante*	193.0501	193.0504	1.7	177.0184, 133.0322, 107.0157
9	*p*-coumaric acid	C_9_H_8_O_3_	3.58	*Triumph*	163.0395	163.0394	1.0	119.0521
				*Rojo Brillante*		163.0394	0.5	145.0258, 117.0308, 93.0340
10	Sinapic acid	C_11_H_12_O_5_	3.91	*Triumph*	223.0606	223.0609	1.3	193.0521, 149.0620, 121.0259
				*Rojo Brillante*	223.0606	223.0608	0.9	193.0490, 191.0334, 121.0294
11	Chlorogenic acid	C_16_H_18_O_9_	2.41	*Triumph*	353.0873	353.0873	5.4	307.0836, 191.0544, 179.0359
	Other/Hydroxybenzaldehydes							
12	Protocatechuic aldehyde	C_7_H_6_O_3_	2.53	*Triumph*	137.0239	137.0243	2.9	121.0268, 109.0289
	Tyrosols							
13	Tyrosol	C_8_H_10_O_2_	5.47	*Triumph*	137.0603	137.0604	0.7	107.0523, 105.0356
				*Rojo Brillante*	137.0603	137.0601	1.5	107.0523, 105.0356
	Flavonoids/Dihydrochalcones							
14	Phloretin	C_15_H_14_O_5_	5.35	*Rojo Brillante*	273.0763	273.0758	1.8	255.0659, 167.0377, 121.0623
15	Phloridzin	C_21_H_24_O_10_	4.60	*Rojo Brillante*	435.1291	435.1299	1.7	273.0761, 151.0047, 125.0239
	Flavonoids/Flavanols							
16	Epicatechin	C_15_H_14_O_6_	2.45	*Triumph*	289.0712	289.0707	1.6	179.0369, 151.0414, 109.0287
				*Rojo Brillante*	289.0712	289.0716	1.4	179.0369, 151.0414, 109.0287
17	Epigallocatechin	C_15_H_14_O_7_	1.67	*Triumph*	305.0661	305.0661	0.1	287.0493, 179.0411, 121.0336
18	Epigallocatechin 7-O-glucuronide	C_21_H_22_O_13_	3.83	*Rojo Brillante*	481.0982	481.0985	0.6	447.0999, 313.0568, 165.0591
	Flavonoids/Flavanones							
19	Naringenin	C_15_H_12_O_5_	6.20	*Rojo Brillante*	271.0606	271.0619	2.0	177.0160, 151.0033, 119.0497
20	Naringenin 7-O-glucuronide	C_21_H_20_O_11_	4.20	*Triumph*	447.0927	447.0935	1.7	269.0468, 255.0289, 227.0318
21	Luteolin	C_15_H_10_O_6_	2.66	*Triumph*	285.0399	285.0401	0.8	229.0176, 201.0266, 150.9992
				*Rojo Brillante*	285.0399	285.0398	0.3	201.0185, 177.0167, 153.0184
	Flavonoids/Flavonols							
22	Kaempferol	C_15_H_10_O_6_	3.99	*Triumph*	285.0399	285.0401	0.8	165.0232, 153.0147, 150.9992
				*Rojo Brillante*	285.0399	285.0398	0.3	165.0125, 153.0184, 133.0365
23	Kaempferol 3-O-galactoside	C_21_H_20_O_11_	4.20	*Triumph*	447.0927	447.0935	1.7	311.0517, 285.0447, 255.0289
				*Rojo Brillante*	447.0927	447.0927	0.2	285.0412, 255.0306, 151.0020
24	Kaempferol 3-O-glucoside	C_21_H_20_O_11_	4.20	*Triumph*	447.0927	447.0935	1.7	311.0517, 285.0447, 255.0289
				*Rojo Brillante*	447.0927	447.0927	0.2	255.0306, 151.0020
25	Kaempferol 3-O-acetyl-glucoside	C_23_H_22_O_12_	4.87	*Rojo Brillante*	489.1033	489.1012	4.2	490.1111, 327.0491
26	Quercetin	C_15_H_10_O_7_	5.52	*Triumph*	301.0348	301.0349	0.4	283.0324, 165.0098, 151.0087
				*Rojo Brillante*	301.0348	301.0355	3.1	175.0043, 151.0044, 137.0245
27	Quercetin 3-O-rhamnoside	C_21_H_20_O_11_	4.20	*Triumph*	447.0927	447.0935	1.7	285.0341
28	Myricetin	C_15_H_10_O_8_	3.11	*Rojo Brillante*	317.0297	317.0297	0.1	193.0136, 165.0182, 151.0029
29	Myricetin 3-O-glucoside	C_21_H_20_O_13_	3.33	*Rojo Brillante*	479.0826	479.0838	2.6	219.0306, 125.0249
30	Myricetin 3-O-rhamnoside	C_21_H_20_O_12_	3.80	*Rojo Brillante*	463.0877	463.0873	0.8	301.0343, 245.0072, 151.0017
31	Isorhamnetin 3-O-glucoside	C_22_H_22_O_12_	4.32	*Rojo Brillante*	477.1033	477.0994	8.2	285.0368, 161.0352, 125.0228

**Table 2:** Individual phenolic compounds quantified in persimmon varieties (mg/100 g fruit pulp).

Phenolic Compounds	*Triumph* Variety (*n* = 10)	*Rojo Brillante* Variety (*n* = 10)
*Hydroxybenzoic acids*		
Gallic acid	0.033 ± 0.035 ^a^	0.953 ± 0.344 ^b^
Vanillic acid	0.021 ± 0.016 ^a^	0.056 ± 0.013 ^b^
*p*-Hydroxybenzoic acid	0.119 ± 0.030 ^a^	0.109 ± 0.039 ^a^
Protocatechuic acid	0.004 ± 0.002 ^a^	0.013 ± 0.010 ^b^
Syringic acid	ND ^a^	0.037 ± 0.024 ^b^
Ellagic acid	ND ^a^	0.327 ± 0.173 ^b^
3,4-Dimethoxybenzoic acid	0.036 ± 0.048 ^a^	0.054 ± 0.025 ^a^
*Hydroxycinnamic Acids*		
Caffeic acid	0.078 ± 0.069 ^a^	0.046 ± 0.060 ^a^
Dimethyl caffeic acid	ND ^a^	0.022 ± 0.001 ^b^
Ferulic acid	0.008 ± 0.003 ^a^	0.017 ± 0.008 ^b^
*p*-coumaric acid	0.088 ± 0.046 ^a^	0.113 ± 0.055 ^a^
4-Methoxycinnamic	0.001 ± 0.001 ^a^	0.002 ± 0.002 ^a^
Sinapic acid	0.002 ± 0.001 ^a^	0.002 ± 0.001 ^a^
Chlorogenic acid	0.007 ± 0.001 ^a^	ND ^a^
*Tyrosols*		
Tyrosol	0.020 ± 0.017	0.038 ± 0.021 ^a^
*Flavonols*		
Quercetin	0.005 ± 0.001	0.007 ± 0.006 ^a^
*Others*		
Pyrogallol	0.001 ± 0.001 ^a^	0.028 ± 0.009 ^b^

ND: not detected; different letters within the same phenolic compounds indicate statistically significant differences (*p* < 0.05).

**Table 3:** Statistical analysis relating to antioxidant activity as measured by DPPH, ABTS, and FRAP assays, and total phenolics as measured by the Folin method in *Rojo Brillante* and *Triumph* varieties.

Method	Equation	Sample	*n*	Mean (SD)
ABTS	y = 130.94x + 4.6699	*Rojo Brillante*	10	6.572 (1.164)
(µmol TE/G)	r^2^ = 0.9995	*Triumph*	10	1.484 (0.249)
DPPH	y = 0.1706x + 6.7611	*Rojo Brillante*	10	2.417 (0.200)
(µmol TE/G)	r^2^ = 0.9958	*Triumph*	10	0.492 (0.039)
FRAP	y = 0.0043x +0.0333	*Rojo Brillante*	10	0.731 (0.178)
(µmol TE/G)	r^2^ = 0.9943	*Triumph*	10	0.242 (0.057)
TP	y = 0.0915x + 0.0931	*Rojo Brillante*	10	380.786 (158.539)
(µg GAE/G)	r^2^ = 0.9991	*Triumph*	10	81.568 (34.989)

SD: standard deviation; ABTS: 2-2′-azino-bis (3-ethylbenzthiazoline-6-sulphonic acid) radical method; DPPH: 1,1-diphenyl-2-picrylhydrazyl radical method; FRAP: ferric reducing antioxidant power assay; TP: Total phenolics; TE: Trolox equivalent; GAE: Gallic acid equivalent.

## Supplementary Materials

The following are available online at https://www.mdpi.com/2076-3921/10/1/31/s1, Figure S1: Chromatogram of a Rojo Brillante sample, chromatogram extracted from vanillic acid and spectrum of vanillic acid, Figure S2: UPLC-QTOF-MS chromatogram Rojo Brillante sample (a) Triumph sample (b), Table S1: Quality parameters for the chromatographic determination of phenolic compounds in persimmon, Table S2: Precision parameters of the method used to determine individual phenolic compounds in persimmon, Table S3: Statistical analysis relating to antioxidant activity as measured by DPPH, ABTS and FRAP assays, and total phenolic compound content as measured by the FOLIN method.
