# Supplementary material for: Differences in the Phenolic Profile by UPLC Coupled to High Resolution Mass Spectrometry and Antioxidant Capacity of Two Diospyros kaki Varieties
Source: Antioxidants (Basel). 2020 Dec 30;10(1):31. doi: 10.3390/antiox10010031 (PMC7823481; doi:10.3390/antiox10010031)
Supplement: Supplementary file 1 [file antioxidants-10-00031-s001.pdf]

Supplementary Materials:

Table S1. Quality parameters for the chromatographic determination of phenolic compounds in persimmon.

Phenolic compounds	Linear range (ppm)	$R_t \pm (\sigma_n\text{-}1)$	Linear equation	R^2	LOD (mg/L)	LOQ (mg/L)
Phenolic acids / Hydroxybenzoic Acids						
Gallic Acid	0.10 - 40	1.25 ± 0.018	y = 62.009x + 17.032	0.998	0.995	3.316
Vanillic acid	0.10 – 40	2.88 ± 0.006	y = 40.782x + 19.537	0.969	1.116	3.719
p-Hydroxybenzoic acid	0.10 – 40	2.55 ± 0.006	y = 25.438x + 14.870	0.990	2.250	7.499
Protocatechuic acid	0.10 – 40	1.89 ± 0.014	y = 62.694x + 5.146	0.995	0.443	1.476
Syringic acid	0.10 – 40	3.01 ± 0.005	y = 72.910x + 46.419	0.985	1.521	5.071
Ellagic acid	0.10 – 40	3.69 ± 0.027	y = 6.943x + 19.840	0.986	5.937	19.789
3,4-Dimethoxybenzoic acid	0.10 – 40	5.50 ± 0.019	y = 33.516x + 90.999	0.987	6.377	21.258
Phenolic acids / Hydroxycinnamic Acids						
Caffeic acid	0.10 – 40	2.83 ± 0.004	y = 157.049x + 83.152	0.990	2.300	7.666
Dimethyl caffeic acid	0.10 – 40	5.02 ± 0.001	y = 73.505x + 23.303	0.991	1.148	3.828
Ferulic acid	0.10 – 40	3.90 ± 0.003	y = 95.024x + 22.681	0.996	0.384	1.282
p-cumaric acid	0.10 – 40	3.54 ± 0.040	y = 60.241x + 38.482	0.992	2.064	6.879
4-Methoxycinnamic acid	0.10 – 40	5.89 ± 0.066	y = 24.461x + 1.204	0.999	0.099	0.329
Sinapic acid	0.10 – 40	3.91 ± 0.015	y = 213.624x + 8.133	0.997	0.082	0.273
Chlorogenic acid	0.10 – 40	2.41 ± 0.001	y = 165.169x – 6.908	0.997	0.610	2.033
Tyrosols						
Tyrosol	0.10 – 40	5.47 ± 0.041	y = 6.851x + 5.560	0.962	2.385	7.951
Flavonoids / Flavonols						
Quercetin	0.10 – 40	5.52 ± 0.003	y = 509.550x + 159.841	0.990	1.220	4.066
Others						
Pyrogallic acid	0.10 – 40	1.27 ± 0.097	y = 41.508x + 23.640	0.993	1.937	6.455

R_t: retention time; σ_n: standard deviation; R: correlation coefficient; LOD: Limit of detection; LOQ: Limit of quantification.

Table S2. Precision parameters of the method used to determine individual phenolic compounds in persimmon.

Phenolic compounds	n	Mean area + $(\sigma_n\text{-}1)$	S^2	CV (%)
Gallic Acid	10	516.4 ± 67.3	0.46	1.30
p-Hydroxybenzoic acid	10	171.9 ± 23.8	2.75	1.38
Caffeic acid	10	108.8 ± 7.21	0.51	0.66
Ferulic acid	10	65.5 ± 6.41	5.85	0.97
p-coumaric acid	10	89.8 ± 2.37	0.73	0.26

σ_n: Standard deviation; S^2: Variance; CV: Coefficient of variation.

Table S3. Statistical analysis relating to antioxidant activity as measured by DPPH, ABTS and FRAP assays, and total phenolic compound content as measured by the FOLIN method.

Antioxidant activity method	Equation	Correlation coefficient (r) (n=10)	Concentration range Trolox
**TPC (700 nm)**	**y = 0.0915 x + 0.092 a**	**0.991**	**0.5-7.5 ppm**

**ABTS•+ (734 nm)**	**y = 0.176 x + 6.671 b**	**0.995**	**10 – 500 μM**
**DPPH (515 nm)**	**y = 130.9 x + 4.889 b**	**0.999**	**0.05 – 0.5 mM**
**FRAP (593 nm)**	**y = 0.041 x + 0.033 b**	**0.994**	**10 – 300 μM**

a y = bx + a, y = absorbance (700 nm), x = Gallic acid concentration. b y = bx + a, y = percentage inhibition, x = Trolox concentration.

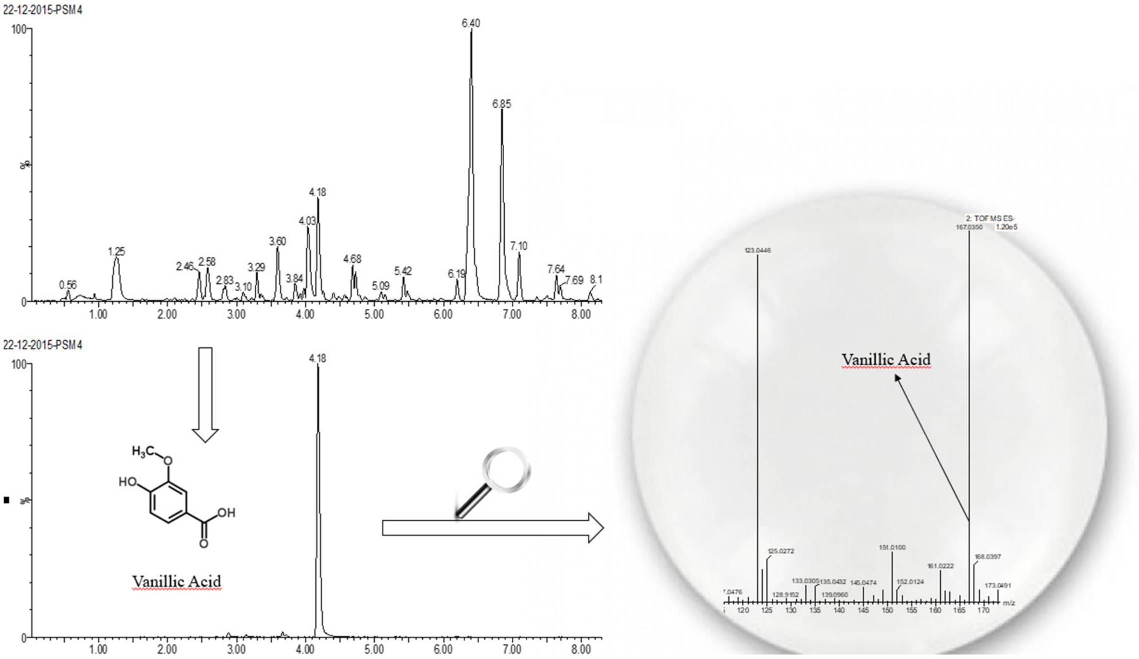

Figure S1. Chromatogram of a *Rojo Brillante* sample, chromatogram extracted from vanillic acid and spectrum of vanillic acid.

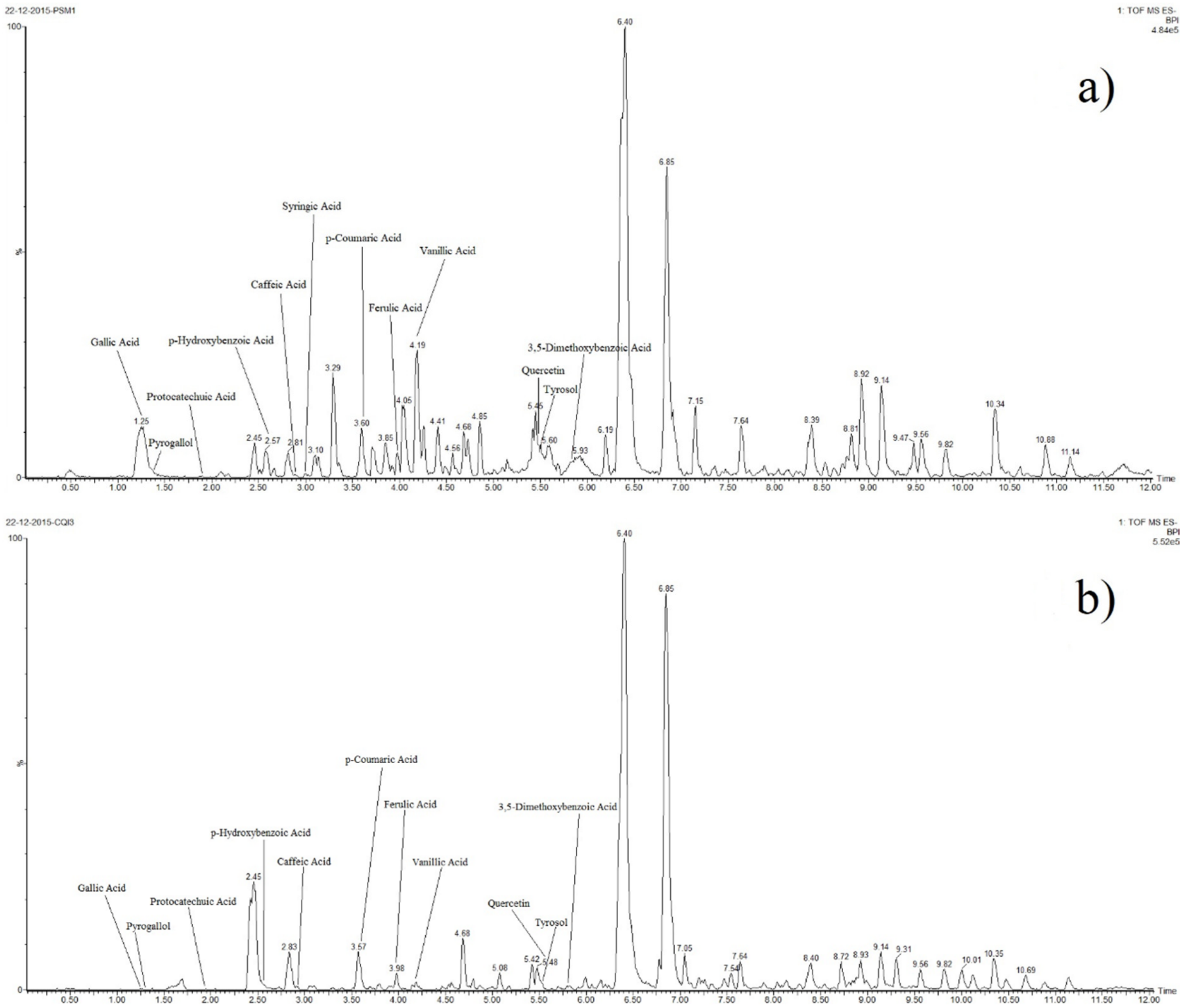

Figure S2. UPLC-QTOF-MS chromatogram *Rojo Brillante* sample (a) *Triumph* sample (b).
